# Circulating RKIP and pRKIP in Early-Stage Lung Cancer: Results from a Pilot Study

**DOI:** 10.3390/jcm13195830

**Published:** 2024-09-29

**Authors:** Roberto Gasparri, Massimo Papale, Angela Sabalic, Valeria Catalano, Annamaria Deleonardis, Federica De Luca, Elena Ranieri, Lorenzo Spaggiari

**Affiliations:** 1 Department of Thoracic Surgery, European Institute of Oncology (IEO), IRCCS, 20141 Milan, Italy; roberto.gasparri@ieo.it (R.G.); lorenzo.spaggiari@ieo.it (L.S.); 2 Unit of Clinical Pathology, Department of Laboratory Diagnostics, University Hospital “Policlinico Foggia”, 71122 Foggia, Italy; 3 Unit of Clinical Pathology, Advanced Research Center on Kidney Aging (A.R.K.A.), Department of Medical and Surgical Sciences, University of Foggia, Viale Luigi Pinto, 71122 Foggia, Italy; valeria.catalano@unifg.it (V.C.); federica.deluca@unifg.it (F.D.L.); elena.ranieri@unifg.it (E.R.); 4 Nephrology, Dialysis and Transplantation Unit, Department of Precision and Regenerative Medicine and Ionian Area (DiMePRe-J), University of Bari “Aldo Moro”, 70121 Bari, Italy; annamaria.deleonardis@uniba.it; 5 R&D Unit, Fluidia s.r.l., 71122 Foggia, Italy; 6 Department of Oncology and Hemato-Oncology, University of Milan, 20141 Milan, Italy

**Keywords:** lung cancer, biomarkers, RKIP, pRKIP, urine, serum

## Abstract

**Background:** Lung cancer (LC) is the leading cause of cancer-related deaths. Although low-dose computed tomography (LD-CT) reduces mortality, its clinical use is limited by cost, radiation, and false positives. Therefore, there is an urgent need for non-invasive and cost-effective biomarkers. The Raf Kinase Inhibitor Protein (RKIP) plays a crucial role in cancer development and progression and may also contribute to regulating the tumor–immune system axis. This protein has recently been described in biological fluids. Therefore, we conducted a pilot case–control study to assess RKIP and phosphorylated RKIP (pRKIP) levels in the urine and blood of LC patients. **Methods:** A novel enzyme linked immunosorbent assay (ELISA) assay was used to measure RKIP and pRKIP levels in urine and blood samples of two cohorts of LC patients and healthy controls (HSs). Furthermore, the biomarkers levels were correlated with tumor characteristics. **Results:** Serum, but not urine, levels of RKIP were significantly elevated in LC patients, distinguishing them from low- and high-risk healthy subjects with 93% and 74% accuracy, respectively. The RKIP/pRKIP ratio (RpR score) showed an accuracy of 90% and 79% in distinguishing LC patients from HS and HR-HS, respectively. Additionally, the RpR score correlated better with dimension, stage, and lymph node involvement in the tumor group. **Conclusions:** The serum RKIP and pRKIP profile may be a promising novel biomarker for early-stage LC.

## 1. Introduction

Lung cancer remains the leading cause of cancer-related deaths, with over 1.8 million deaths worldwide [1]. It is a latent and asymptomatic disease, with only 15% of lung cancer patients surviving five years after diagnosis, since approximately 70% of patients have advanced disease at the time of diagnosis [2]. Tobacco use remains the most significant risk factor [1].

Studies have shown that lung cancer causes more deaths than breast and colorectal cancers combined [3]. This may be partly due to the current lack of an effective screening test for early-stage lung cancer detection. According to the literature, the National Lung Screening Trial (NLST) demonstrated a 20% reduction in lung cancer mortality by screening high-risk patients with low-dose chest CT (LD-CT). However, the rate of false positives and overdiagnosis has highlighted the need for new and more reliable screening tests to improve early detection and increase overall patient survival [3,4]. In this context, research has made significant efforts to identify non-invasive and cost-effective biomolecules in biological samples that could be useful for screening high-risk populations for acute, chronic, and non-communicable diseases [5,6,7,8,9,10,11].

Among tumor-related biomarkers, proteins are particularly interesting because they are relatively stable and are the biological end-points responsible for most cellular functions, often regulated by post-translational modifications (PTMs) [12]. A key prerequisite for identifying reliable biomarkers could be evaluating molecules with a well-established role in the pathogenesis of a disease. In this context, the Raf Kinase Inhibitor Protein (RKIP) is a crucial modulator of intracellular signaling pathways, finely regulated at both transcriptional [13] and post-transcriptional levels [14,15,16]. The RKIP plays a significant role in the development and progression of various cancers [17,18].

Although it was initially identified as an endogenous inhibitor of the Raf kinase pathway [19], several other functions have since been reported. For example, RKIP inhibition through Protein Kinase C (PKC) phosphorylation determines both the overall activation of G-Protein Coupled Receptors (GPCRs) and the hyperstimulation of the MAP kinase pathway [20]. Additionally, RKIP downregulation activates many upstream kinases involved in NK-kB and STAT 3 signaling, which, in turn, modulate the expression of several genes related to cell growth, apoptosis, survival, and differentiation [21,22].

Furthermore, recent studies have suggested a novel role of the RKIP as a negative regulator of the tumor microenvironment [23]. Yang et al. [24] reported an inverse correlation between the expression of the cluster of differentiation 44 (CD44), a well-known tumor marker of gastric cancer, and RKIP expression, which suggests an RKIP-mediated inhibition of initial tumor development. Furthermore, the RKIP appears to control tumor-associated macrophage (TAM) infiltration by reducing the expression of chemotactic factors such as chemokine ligand 5 (CCL5) [25].

Given the growing body of data showing a wide variety of functions of the RKIP, it is now clear that this molecule plays a far more complex role than the mere inhibition of Raf kinase.

The RKIP, also known as Phosphatidylethanolamine-binding protein 1 (PEBP1), has been recently identified in blood [26,27], cerebrospinal fluid (CSF) [28,29], and urine [30].

While reduced levels of urine RKIP appear to reflect its downregulation in clear cell renal cancer, a trend of increased PEBP1 in the CSF of Alzheimer’s patients compared to controls was observed, although its role remains unclear. In contrast, the administration of highly anti-inflammatory monoclonal antibodies induced a significant reduction in plasma RKIP in multiple sclerosis patients, thus suggesting a potential cross-talk between the RKIP and the immune system.

Given the importance of the relationship between the immune system and tumor development in the early stages of disease, along with the relationship between plasma RKIP levels and immune system activation, we set up the first prospective, monocentric case–control pilot study regarding this issue. The aim was to evaluate the behavior of the RKIP and its phosphorylated form (pRKIP) in the urine and blood samples of lung cancer patients to establish their potential role as biomarkers of immune system activation in lung cancer.

## 2. Materials and Methods

### 2.1. Study Design and Patient Selection

The present study was a prospective, single-center, case–control study conducted on urinary and serum samples from two consecutive and distinct cohorts of lung cancer patients and healthy controls. Samples were collected by the Thoracic Surgery Division of the European Institute of Oncology, Milan, Italy. The study protocol was approved by the Ethics Committee, n. R846/18-IEO890. The first cohort included 42 individuals: 21 lung cancer patients (LC) undergoing surgery at the Thoracic Surgery Division of European Institute of oncology (IEO), and 21 high-risk healthy subjects (HR-HSs). These high-risk subjects included heavy smokers, individuals with non-cancer related pulmonary disease, those with a family history of lung cancer, or those with occupational exposure to risk factors, aged 60–80 years (Table 1). All subjects underwent an LDCT scan to confirm the presence of malignant nodules in the lung cancer group and negativity (the absence of oncologically suspicious nodules) in the HR-HS group. Lung cancer diagnosis and staging were confirmed by histological examination according to TNM^8 Ed. (Table 2). None of the subjects had a history of cancer in the previous five years, nor had they received radio/chemotherapy. The second cohort included 18 LC patients and 21 HR-HSs aged 50–70 years. The inclusion criteria adopted were the same as described for the first cohort. An additional independent control group of 11 chest CT/X-ray-negative healthy subjects without any risk factors for lung cancer and no family history or symptoms was also recruited (HS group). At registration, all subjects were fully informed, signed the study-specific informed consent form, and completed a clinical questionnaire. The analysis of the RKIP and pRKIP was performed on urine samples from the first cohort and serum samples from the second cohort. Clinical characteristics of the enrolled patients are shown in Table 1.

The sample size was calculated by a two-sample *t*-test (the difference in two independent means). A sample of 21 LC patients and 21 controls achieved 80% power to detect a difference of −45.2 ng/mg/g of the RKIP between the null hypothesis (both group means are 62.3 ng/mg/g of RKIP) and the alternative hypothesis (the mean for the lung cancer group is 17.1 ng/mg/g) with estimated group standard deviations of 67.5 and 13.1 and a significance level (alpha) of 0.05 using a two-sided two-sample *t*-test.

### 2.2. Sample Collection and Analysis

A 50 mL urine sample was collected from each patient in the morning, centrifuged at 1000× *g* for 10 min at 4 °C, aliquoted, and immediately frozen at −80 °C until use. Blood samples were collected by standard phlebotomy. The serum was prepared by leaving blood in the tubes for at least 30 min at room temperature (RT) to allow blood clotting, followed by centrifugation at 1000× *g* for 10 min at RT. The serum was removed immediately after centrifugation, leaving 0.5 cm leftover to avoid disturbing the serum–clot interface, and then aliquoted and stored at −80 °C until use. Urinary and blood levels of the RKIP and Ser153 phosphorylated-RKIP were measured using sandwich experimental ELISA assays developed by Fluidia s.r.l. (Foggia, Italy). Briefly, this assay allows the parallel and quantitative assessment of both native and phosphorylated RKIP, potentially, in any sample type. To test the RKIP and pRKIP in urine samples, 100 μL of diluted samples and 100 μL of reconstituted standard were loaded, in duplicate, into two series of wells coated with a specific capture antibody. After incubation, the wells were washed thoroughly in a washing solution and subsequently incubated with Detection Reagent A or Detection Reagent B, which were designed to recognize the immunocomplexes RKIP-RKIPAb or pRKIP-pRKIPAb, respectively. After a series of washing steps, the plates were incubated with Detection Reagent C. The amount of the antigen–antibody complex was revealed by adding a chromogenic substrate, and the optical density was measured at 450 nm. RKIP and pRKIP levels in urine samples were normalized to urine creatinine (uCr), with RKIP and pRKIP concentrations expressed as ng/mg uCr. Urinary and serum excretion of the RKIP and pRKIP as well as the RKIP/pRKIP ratio (R/pR score) were calculated for each patient and used, either individually or in combination, for statistical analysis.

### 2.3. Statistical Analysis

The analysis of the RKIP and pRKIP was performed where appropriate on urine and serum samples, in the first and second cohorts, respectively. All experiments were repeated three times. Statistical analysis was performed using Microsoft Excel (https://www.microsoft.com, Microsoft Inc., Redmond, WA, USA). Data are presented as the M ± SD or median as appropriate. Comparisons between groups were conducted using Student’s *t*-test. *p* < 0.05 was considered statistically significant. A receiver operating characteristic (ROC) curve analysis was used to validate the association between the RKIP, pRKIP, or RKIP/pRKIP (RpR) ratio and LC. The cut-off value was set by the percentile method to determine the diagnostic power of each assay according to the area under the ROC curve.

## 3. Results

### 3.1. Evaluation of RKIP and pRKIP Levels in Urine Samples

**Figure 1:**
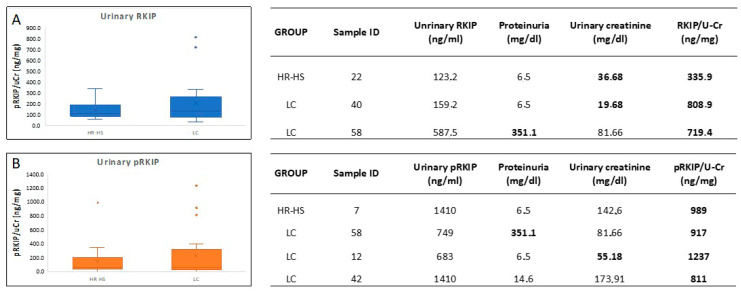
RKIP and pRKIP urinary excretion in the urine of HR-HSs and LC patients. Urinary RKIP (**A**) and pRKIP (**B**) showed large variability in HR-HS (138 ± 78 ng/mg/uCr for RKIP and 142 ± 219 ng/mg/uCr for pRKIP) and LC (203 ± 205 ng/mg/uCr for RKIP and 226 ± 345 ng/mg/uCr for pRKIP) groups. Furthermore, the differences between LC patients and HR-HSs were not statistically significant (*p*-value 0.09). Table in the panel A and table in the panel B show proteinuria and urine creatinine of the patients with a higher titer of the RKIP and pRKIP, respectively. The higher levels of proteinuria and/or the lower of urinary creatinine are represented in bold.

To establish whether urinary RKIP could successfully identify LC patients, we first analyzed RKIP levels in urine samples from early-stage lung cancer patients matched with a high-risk control (HR-HS group). HR-HSs and LC patients showed 138 ± 78 ng/mg/uCr and 203 ± 205 ng/mg/uCr urinary RKIP, respectively. We measured a large variability of RKIP levels in both groups, ranging from 57 to 336 ng/mg/uCr (median 104 ng/mg/uCr) in the HR-HS group and from 31 to 809 ng/mg uCr (median 133 ng/mg/uCr) in the LC group (Figure 1A and Table S1). Although the LC group showed a trend of increased urinary RKIP, the differences between the groups were not statistically significant (*p*-value 0.09). Furthermore, we assessed the urinary concentration of phosphorylated RKIP (pRKIP) in the same cohort. Again, there was a large intra-group variability (142 ± 219 ng/mg/uCr for HR-HS and 226 ± 345 ng/mg/uCr for LC), with values ranging between 0 to 989 ng/mg/uCr in the HR-HS group and from 0 and 1237 ng/mg/uCr in the LC group (Figure 1B and Table S2), with no statistically significant difference between the groups. Notably, clinical data analysis showed that some patients with a higher urinary RKIP titer also had proteinuria or reduced urine creatinine excretion (Tables in Figure 1).

### 3.2. Evaluation of RKIP and pRKIP Levels in Serum Samples

**Figure 2:**
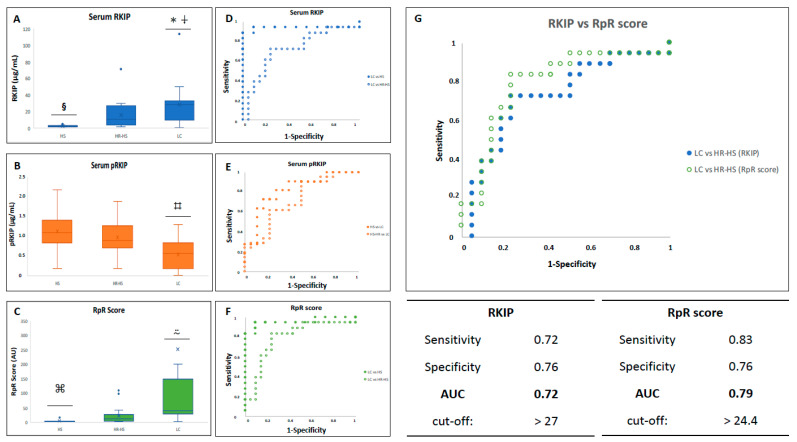
Serum levels of RKIP and pRKIP assayed by sandwich ELISA. Total (**A**) and ser-153 phosphorylated (**B**) serum RKIP assayed by sandwich ELISA. Ratio between total RKIP and pRKIP is shown in panel (**C**); ✲ = *p*-value 3.3883 × 10^−7^ LC vs. HS patients; ⍖ = *p*-value < 0.05 LC vs. HR-HS patients; § = *p*-value < 0.001 HSs vs. HR-HSs; ⌗ = *p*-value < 0.005 LC patients vs. HSs and HR-HSs; ⍨ = *p*-value < 0.01 LC patients vs. HSs and HR-HSs; ⌘ = *p*-value < 0.01 HSs vs. HR-HSs. (**D**) AUC obtained by measuring total serum RKIP in LC patients vs. HSs (full dots) or HR-HSs (empty dots); (**E**) AUC obtained by measuring serum pRKIP in HS vs. LC patients (dots) or HR-HS vs. LC patients (empty dots); (**F**) AUC (in bold) obtained by measuring serum RKIP/pRKIP ratio in LC vs. HS patients (full dots) or LC vs. HR-HS patients (empty dots); (**G**) Comparative representation of AUC obtained by classifying LC over HR-HS patients by RKIP and RpR score, respectively.

Considering the results obtained from urine samples, we extended the analysis of the RKIP and pRKIP to the blood samples by recruiting a new cohort of LC patients, HR-HSs, and, additionally, a group of low-risk HSs. In the blood, we measured 23.6 ± 11.8 μg/mL total RKIP in the LC group, 15.7 ± 16.3 μg/mL in the HR-HS group, and 2.4 ± 0.96 μg/mL in the HS group (Figure 2A). RKIP levels ranged from 1 to 37.1 μg/mL (median value 28.13 μg/mL) in the LC group, from 1.8 to 70.8 in the HR-HS group (median 10.4 μg/mL), and from 1.3 and 4.7 μg/mL in the HS group (median 2.2 μg/mL) (Table S3). The differences between the LC group and the other groups were statistically significant (*p*-values LC vs. HS and HR-HS were 3.3883 × 10^−7^ and <0.05, respectively). The differences between the HS and HR-HS groups were also significant (*p*-value < 0.001). By contrast, we assessed higher levels of pRKIP (1.1 ± 0.5 μg/mL) in the HS group (0.2–2.2 μg/mL; median 1.1 μg/mL), intermediate values (1 ± 0.4 μg/mL) in the HR-HS group (0.2–1.9 μg/mL; median 0.9 μg/mL), and lower levels (0.6 ± 0.4 μg/mL) in the LC group (0.1–1.1 μg/mL; median 0.7 μg/mL) (Figure 2B and Table S4). The differences between the LC group and the HS and HR-HS groups were statistically significant (*p*-value < 0.005). The opposite trends in the concentrations of the RKIP and pRKIP in the LC group vs. the HS and HR-HS groups resulted in an increase in the RKIP/pRKIP ratio (RpR score) in the affected patients (Figure 2C). The median RpR scores were 1.8 (1.3–14.9) in the HS group, 11.1 (1.9–108.6) in the HR-HS group, and 36.1 (1.7–370) in the LC group. The differences based on the RpR score were statistically significant even between the LC group and both control groups (*p*-value LC vs. HS < 0.01; LC vs. HR-HS < 0.05) and between HSs and HR-HSs (*p*-value < 0.01). We further calculated the Area Under the Curve (AUC) for the RKIP, pRKIP, and RpR score by applying ROC analysis. Serum RKIP correctly classified LC patients from healthy subjects with 94% sensitivity and 91% specificity (AUC 0.94, accuracy 93%) by using a cut-off value of 3.86 μg/mL (Figure 2D), while it reached 72% sensitivity and 76% specificity (AUC 0.72, accuracy 74%) when the classification model was applied to LC patients vs. HR-HSs (cut-off value: 27 μg/mL). The diagnostic model based on the assessment of serum pRKIP (cut-off value for LC patients < 0.8 μg/mL) showed 72% sensitivity and 82% specificity (AUC 0.81, accuracy 76%) when it was used to classify LC over HS patients. It showed 72% sensitivity vs. 62% specificity (AUC 0.72, accuracy 67%) when it was used to classify LC over HR-HS patients (Figure 2E). Finally, the calculation of the AUC based on the combined analysis of the RKIP and pRKIP demonstrated the best diagnostic performance: the model reached 83% sensitivity and 100% specificity (AUC 0.95, accuracy 90%) when it was used to classify LC vs. HS patients, and 83% sensitivity and 76% specificity (AUC 0.79, accuracy 79%) when it was used to classify LC vs. HR-HS patients (Figure 2F). Overall, the ROC curve based on the RpR score provided more accurate diagnostic performance than using the RKIP alone (AUC 0.79 vs. 0.72—Figure 2G).

### 3.3. Analysis of Serum RKIP and pRKIP Levels According to Tumor Characteristics

**Figure 3:**
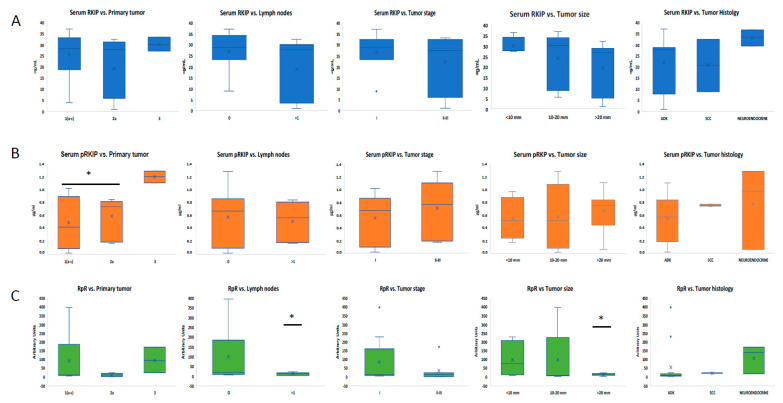
Analysis of RKIP, pRKIP, and RpR scores according to LC characteristics. Stratification of LC patients according to RKIP (**A**), pRKIP (**B**), and RpR levels (**C**) are represented. * = *p*-value < 0.05 ADK = adenocarcinoma; SCC = squamous cell carcinoma.

To assess whether RKIP levels could vary in the LC group based on tumor characteristics, patients were stratified according to primary tumor classification, the presence of lymph node metastases, stage, tumor size, and histological features. Correlations were made both by considering the RKIP and pRKIP individually and by relating them by means of the RpR score. Serum RKIP alone did not correlate significantly with any of the parameters tested and there was only a slight increase in patients who did not have lymph node involvement and, regarding tumor type, in patients with the neuroendocrine form (Figure 3A). pRKIP showed a statistically significant increase only in the group of patients with primary tumor classification 3, while no relevant correlations were observed with the other parameters tested (Figure 3B). In contrast, stratifying patients by RpR score revealed statistically significant differences (*p*-value < 0.05) in relation to both tumor size and lymph node involvement (Figure 3C). Specifically, patients with tumors of < 10 mm at diagnosis and/or without lymph node metastases had a significantly higher RpR score than patients with tumors > 20 mm and/or lymph node metastases. The RpR ratio also tended to be higher in stage 1 patients than in stage 2 or 3 patients.

## 4. Discussion

The identification of novel biomarkers for the early diagnosis of lung cancer represents a key challenge in developing a non-invasive and cost-effective screening test, which could improve clinical practice and patient prognosis. To the best of our knowledge, this study is the first to describe the potential utility of blood-based assessment of the RKIP and pRKIP as novel biomarkers for the early identification of NSCLC.

The research was conducted using urinary and serum samples from two independent cohorts: patients diagnosed with lung cancer and high-risk healthy individuals with a negative LDCT for lung cancer. To investigate how the expression levels of the RKIP and pRKIP differ between these groups, we first analyzed the urine samples from the first cohort enrolled (Table 1). We chose urine as the first sample for analysis based on our previous results by Gasparri et al. [31], which demonstrated the diagnostic power of urinary volatile organic compounds (VOCs) for detecting early-stage lung cancer, and Papale et al. [30], who highlighted the potential of assessing urinary RKIP and pRKIP as novel biomarkers for clear cell Renal Cell Carcinoma (ccRCC). Thus, we explored the possibility of translating the results already obtained in kidney cancer to a lung cancer cohort with the aim of further strengthening the diagnostic power of the model based on the VOC profile. The analysis of urinary RKIP/pRKIP in this cohort showed overall overlapping levels of the biomarker between LC patients and matched controls, with a slight increase in RKIP levels in the LC group. However, some patients exhibited unusually higher levels of the biomarker in their urine. A detailed analysis of these patients’ characteristics revealed that some of those with higher urinary RKIP values also had increased proteinuria or reduced urine creatinine (Figure 1), two conditions that may indicate kidney impairment and protein loss. Interestingly, this suggests that the blood concentration of RKIP might be greater than that measured in urine, and that the slight differences in urinary excretion may be partially due to the kidney’s ability to reabsorb most of the filtered RKIP, potentially underestimating the differences between the groups.

Furthermore, RKIP levels in the blood of healthy subjects were recently assessed using a Proximity Extension Assay (PEA), a proteogenomic approach that combines antibody-based immunoassays with polymerase chain reaction [27]. The authors reported that the protein is present in the blood of healthy individuals and does not vary between males and females or over time. However, they did not test it in the blood of LC patients.

Therefore, we decided to expand the research by recruiting a second cohort of LC patients and matched healthy subjects, focusing on blood samples. In the first set of exploratory experiments using indirect ELISA, we observed a statistically significant increase in the total RKIP in the blood of LC patients. Additionally, higher levels of pRKIP were found in the HS group compared to both HR-HS and LC groups, which, in turn, resulted in an overall increase in the RKIP/pRKIP ratio (RpR score) in both at-risk and lung cancer patients (Supporting Figure S1).

To increase the sensitivity of the assay, we re-screened the samples using a more accurate sandwich ELISA. This analysis confirmed the increase in blood RKIP levels and, most importantly, highlighted a trend of reduced phosphorylated RKIP in the LC group, leading to a statistically significant increase in the RpR score in this group. ROC curve analysis of the RKIP and pRKIP demonstrated that blood RKIP alone could correctly classify LC patients compared to low- and high-risk HS subjects with 93% and 74% accuracy, respectively. The pRKIP-based classification model identified LC patients with an accuracy of 76% when compared to the HS group and 72% when compared to the HR-HS group. Finally, the model based on the evaluation of the RKIP/pRKIP ratio showed a 90% diagnostic accuracy for LC when compared to the HS group, and most importantly, up to 79% accuracy when compared to the high-risk group. Interestingly, the patient in the HR-HS group with the highest RKIP value (70.8 µg/mL) developed LC the year following the analysis. This finding further supports the predictive value of this test for the early detection of lung cancer. Furthermore, by stratifying LC patients based on tumor characteristics, we established that the RpR score is inversely proportional to the size of the primary tumor and the presence of lymph node metastases. These data support the idea that the highest levels of circulating RKIP are obtained in the very early stages of disease development. As the disease progresses, there is a significant reduction in RKIP expression in tumor cells that favors metastasis formation and disease progression. This is consistent with reports in the published biomedical literature. In our model, we observed a significant increase in the active form of the RKIP in the blood of early-stage LC patients, along with a concomitant significant reduction in the inactive (phosphorylated) form. This, in turn, led to a significant increase in the RpR ratio, a novel cancer score postulated by Papale et al. [18,30] as a preliminary screening test for detecting lung cancer in high-risk subjects.

We hypothesize that an increase in plasma RKIP may be related to the activation of the immune system in response to cancer cells during the early stages of disease development. In this context, the RKIP could potentially serve as a surrogate biomarker for early-stage lung cancer. Our hypotheses are based on scientific evidence suggesting the crucial role of the RKIP in modulating the interaction between the host immune system and the tumor. Specifically, the RKIP may regulate tumor-associated macrophage (TAM) infiltration [23], inhibit cancer invasion and metastasis by controlling chemokine expression [25], and function as a novel negative regulator of the tumor microenvironment [24]. Furthermore, Bedri et al. [26] provided the first evidence of a correlation between plasma RKIP and the immune system in patients undergoing immune-modulating therapy.

We believe that our data are consistent with the published peer-reviewed biomedical literature, which demonstrates a strong correlation between RKIP downregulation in cancer cells and tumor progression. The increased RKIP levels in the plasma of lung cancer patients may not be directly related to the tumor itself but rather to the regulation of the host immune system’s response to the tumor. This aspect could also help counteract tumor progression, especially in the early stages, as inflammation is a hallmark of cancer and is mediated by immune cells that are attracted to or reside at sites of neoplastic transformation [32,33,34,35]. At these sites, the communication between the tumor and the immune system forms the basis for the disease’s pathophysiology.

### Study Limitations

The present study underlined the potential of the RKIP and pRKIP as surrogate markers capable of distinguishing lung cancer patients from healthy subjects. However, the study is not without certain limitations, which warrant consideration. The research was conducted on a limited number of subjects in a monocentric study. Additionally, it has not yet been possible to determine whether, and how quickly, the removal of the tumor leads to the normalization of blood levels of the biomarker. Unfortunately, the experimental protocol approved by the ethics committee did not include the collection and storage of tissue samples or peripheral blood cells, which prevented us from correlating serum RKIP levels with levels of expression in tumor cells. Furthermore, based on the reported data, it would be interesting to correlate plasma RKIP levels with tumor macrophage infiltration and to verify the exact source of plasma RKIP, particularly in circulating lymphocytes [35]. These aspects will need to be investigated in further studies, and the recruitment of an independent cohort of patients will be essential to more accurately establish the optimal cut-off value of each biomarker, enabling the development of a standardized risk score for early-stage lung cancer.

## 5. Conclusions

In conclusion, our findings reveal that blood RKIP levels surge in the earliest stages of lung cancer, while its phosphorylated counterpart diminishes. Crucially, the RKIP/pRKIP ratio offers a far more precise risk assessment than evaluating either biomarker in isolation. These results represent a pivotal step forward in understanding the potential of blood RKIP and pRKIP as biomarkers for the early diagnosis of lung cancer, paving the way for more effective, non-invasive diagnostic tools that could transform patient outcomes and alter the course of this devastating disease.

## Figures and Tables

**Table 1:** Characteristics of participants.

	COHORT 1				COHORT 2			
	All	Lung Cancer	Healthy Controls	*p*-Value	All	Lung Cancer	Healthy Controls	*p*-Value
Subjects (n)	42 (100)	21 (100)	21 (100)		41 (100)	18 (100)	21 (100)	
Mean Age	65.5 ± 6.3	67.1 ± 6.6	63.9 ± 5.5	0.089	68.0 ± 6.8	69.8 ± 6.7	66.1 ± 6.4	0.083
Sex								
Female	18 (42.9)	10 (47.6)	8 (38.1)		16 (39.0)	11 (61.1)	5 (23.8)	
Male	24 (57.1)	11 (52.4)	13 (61.9)	0.76	24 (58.5)	9 (50.0)	15 (71.4)	0.11
Smoking status								
Current smokers	15 (35.7)	7 (33.3)	8 (38.1)		8 (19.5)	2 (11.1)	6 (28.6)	
Ex-smokers	20 (47.6)	9 (42.9)	11 (52.4)		25 (61.0)	12 (66.7)	13 (61.9)	
Never-smokers	7 (16.7)	5 (23.8)	2 (9.5)	0.60	7 (17.1)	6 (33.3)	1 (4.8)	0.081
Mean Pack-years	51.1 ± 35.3	58.9 ± 35.0	44.6 ± 35.1	0.24	30.5 ± 29.5	30.0 ± 29.1	30.9 ± 30.6	0.93
Commorbidities								
AH ^1^	17 (40.5)	11 (52.4)	6 (28.6)	0.21	19 (46.3)	12 (66.7)	7 (33.3)	0.20
Cardiac disease	4 (9.5)	4 (19.0)	0 (0.0)	0.11	3 (7.3)	0 (0.0)	3 (14.3)	0.23
Metabolic disease ^2^	17 (40.5)	8 (38.1)	9 (42.9)	1.00	12 (29.3)	5 (27.8)	7 (33.3)	0.73
COPD ^3^	4 (9.5)	0 (0.0)	4 (19.0)	0.11	3 (7.3)	0 (0.0)	3 (14.3)	0.23

AH ^1^ = arterial hypertension; metabolic disease; ^2^ = diabetes and dyslipidemia; COPD ^3^ = chronic obstructive pulmonary disease.

**Table 2:** Lung cancer histology.

		COHORT 1	COHORT 2
Histology	Adenocarcinoma	17	13
	Squamous-cell carcinoma	1	2
	Neuroendocrine	3	3
Stage	IA	5	3
	IA2	6	2
	IA3	4	4
	IB	6	2
	IIB	-	3
	IIIA	-	4

## Data Availability

The data presented in this study are available on request from the corresponding authors due to privacy.

## Supplementary Materials

The following supporting information can be downloaded at: https://www.mdpi.com/article/10.3390/jcm13195830/s1, Table S1: mean value of urinary RKIP as well as proteinuria and urinary creatinine recorded for each patient enrolled in phase 1. Table S2: mean value of urinary pRKIP as well as proteinuria and urinary creatinine recorded for each patient enrolled in phase 1. Table S3: mean values of serum RKIP, pRKIP, and RpR score recorded by indirect ELISA for each patient enrolled in phase 2. Table S4: mean values of serum RKIP, pRKIP, and RpR score recorded by sandwich ELISA for each patient enrolled in phase 2. Figure S1: Serum levels of RKIP and pRKIP assessed by indirect ELISA.
