# Supplementary material for: Circulating RKIP and pRKIP in Early-Stage Lung Cancer: Results from a Pilot Study
Source: J Clin Med. 2024 Sep 29;13(19):5830. doi: 10.3390/jcm13195830 (PMC11476948; doi:10.3390/jcm13195830)
Supplement: Supplementary file 1 [file jcm-13-05830-s001.zip › Figure S1.pdf]

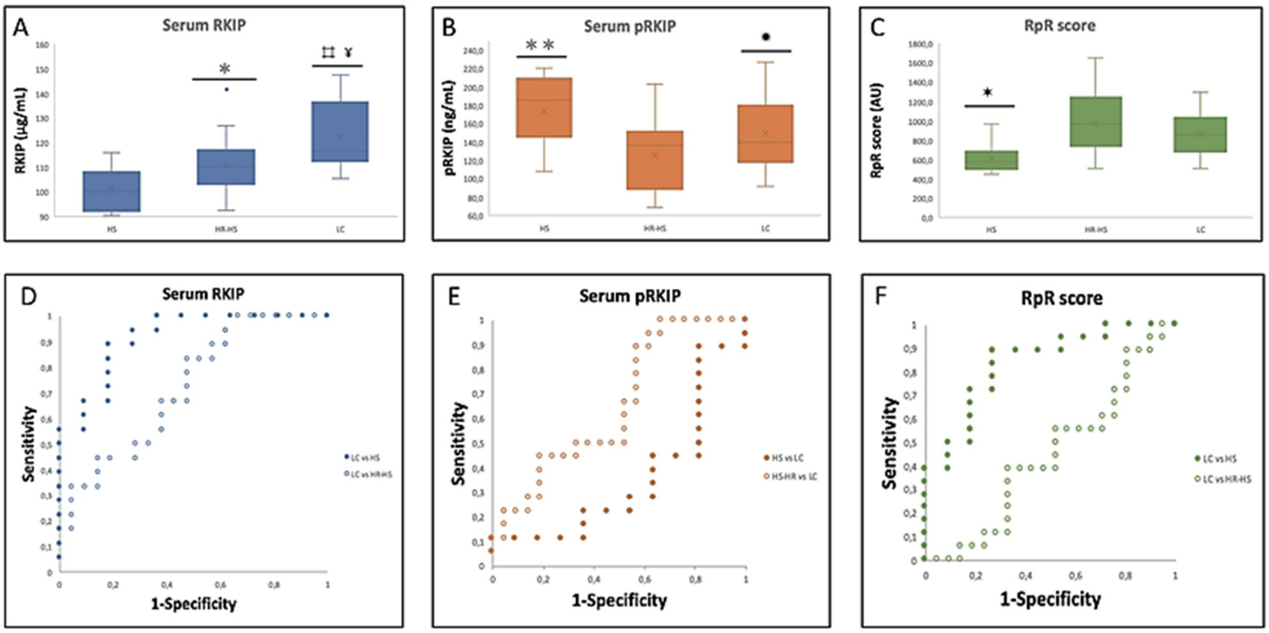

LC vs HS		LC vs HR-HS	
Sensitivity	0.83	Sensitivity	0.83
Specificity	0.82	Specificity	0.55
AUC	0.91	AUC	0.72

HS Vs LC		HR-HS vs LC	
Sensitivity	0.56	Sensitivity	0.56
Specificity	0.82	Specificity	0.33
AUC	0.65	AUC	0.35

LC Vs HS		LC vs HR-HS	
Sensitivity	0.72	Sensitivity	0.72
Specificity	0.82	Specificity	0.24
AUC	0.83	AUC	0.44

Figure S1. Serum levels of RKIP and pRKIP assessed by indirect ELISA

Total (A) and ser-153 phosphorylated (B) serum RKIP assayed by indirect ELISA. Ratio between Total RKIP and pRKIP is shown in panel C; ⌗ = p-value = 1.93704E-07 LC vs HS; ¥ p-value < 0.005 LC vs HR-HS ; ✳ = p-value < 0.005 HS vs HR-HS; ✹ = p-value < 0.05 HR-HS vs LC; ✳ ✳ = p-value < 0.0005 HS-HR vs HS ; ✶ = p-value < 0.005 HS vs HR-HS and LC . D. AUC obtained by measuring total serum RKIP in LC vs HS (solid circles) or HR-HS (empty circles); E. AUC obtained by measuring serum pRKIP in Hs vs LC (solid circles) or HR-HS vs LC (empty circles); F. AUC obtained by measuring serum RKIP/pRKIP ratio in Hs vs LC (solid circles) or HR-HS vs LC (empty circles);
