# Supplementary material for: Circulating RKIP and pRKIP in Early-Stage Lung Cancer: Results from a Pilot Study
Source: J Clin Med. 2024 Sep 29;13(19):5830. doi: 10.3390/jcm13195830 (PMC11476948; doi:10.3390/jcm13195830)
Supplement: Supplementary file 1 [file jcm-13-05830-s001.zip › Table S1.pdf]

GROUP	Sample ID	Mean Unrinary RKIP (ng/ml)	Proteinuria (mg/dl)	Urinary creatinine (mg/dl)	Mean RKIP/U-Cr (ng/mg)
HR-HS	8	77	6.5	122.18	63
HR-HS	9	178	6.5	149.84	118
HR-HS	10	187	6.5	67.31	278
HR-HS	11	92	9.9	82.56	111
HR-HS	13	131	6.5	150.83	87
HR-HS	14	140	10.1	168.34	83
HR-HS	15	145	9.7	93.88	154
HR-HS	16	103	16.4	180.88	57
HR-HS	19	52	6.5	21.87	236
HR-HS	20	86	6.5	91.93	93
HR-HS	21	76	6.5	34.83	218
HR-HS	23	98	6.5	76.83	128
HR-HS	24	103	6.5	39.99	257
HR-HS	25	118	6.5	91.9	129
HR-HS	26	73	7.9	72.83	101
HR-HS	27	111	15.9	155.21	72
HR-HS	33	121	11.1	130.53	93
HR-HS	12	124	6.5	119.02	104
HR-HS	22	123	6.5	36.68	336
HR-HS	7	120	6.5	142.6	84
HR-HS	32	128	9.5	138.25	93
LC	6	76	6.5	242.97	31
LC	18	115	13.4	56.93	202
LC	30	145	6.5	109.245	133
LC	34	136	6.5	40.63	334
LC	38	88	6.5	135.83	65
LC	40	159	6.5	19.68	809
LC	41	98	6.5	86.65	113
LC	48	87	6.5	175.54	50
LC	50	127	17.4	82.31	154
LC	52	105	6.5	135.19	78
LC	55	110	6.5	86.8	127
LC	56	140	6.5	52.49	267
LC	57	117	8.8	159.62	73
LC	58	587	351.1	81.66	719
LC	59	90	6.5	124.79	72
LC	60	133	6.5	71.38	187
LC	63	128	9	183.76	69
LC	43	155	6.5	55.18	282
LC	35	244	7	93.19	261
LC	42	167	14.6	173.91	96

LC	36	196	20.3	139.19	141

Table S1. Mean vale of urinary RKIP as well as proteinuria and urinary creatinine recorded for each patient enrolled in phase 1.
