# Supplementary material for: Circulating RKIP and pRKIP in Early-Stage Lung Cancer: Results from a Pilot Study
Source: J Clin Med. 2024 Sep 29;13(19):5830. doi: 10.3390/jcm13195830 (PMC11476948; doi:10.3390/jcm13195830)
Supplement: Supplementary file 1 [file jcm-13-05830-s001.zip › Table S2.pdf]

GROUP	Sample ID	Mean Urinary pRKIP (ng/ml)	Proteinuria (mg/dl)	Urinary creatinine (mg/dl)	Mean pRKIP/U-Cr (ng/mg)
HR-HS	8	80	6.5	122.18	65.5
HR-HS	9	46	6.5	149.84	30.7
HR-HS	10	43	6.5	67.31	63.9
HR-HS	11	36	9.9	82.56	43.6
HR-HS	13	30	6.5	150.83	19.9
HR-HS	14	n.d.	10.1	168.34	n.d.
HR-HS	14	16	9.7	93.88	17.0
HR-HS	16	43	16.4	180.88	23.8
HR-HS	19	76	6.5	21.87	347.5
HR-HS	20	30	6.5	91.93	32.6
HR-HS	21	46	6.5	34.83	132.1
HR-HS	23	26	6.5	76.83	33.8
HR-HS	24	69	6.5	39.99	172.5
HR-HS	25	90	6.5	91.9	97.9
HR-HS	26	23	7.9	72.83	31.6
HR-HS	27	53	15.9	155.21	34.1
HR-HS	33	63	11.1	130.53	48.3
HR-HS	12	327	6.5	119.02	274.5
HR-HS	22	85	6.5	36.68	232.6
HR-HS	7	1410	6.5	142.6	988.9
HR-HS	32	402	9.5	138.25	291.0
LC	6	43	6.5	242.97	17.7
LC	18	111	13.4	56.93	195.0
LC	30	39	6.5	109.245	35.7
LC	34	16	6.5	40.63	39.4
LC	30	26	6.5	135.83	19.1
LC	40	59	6.5	19.68	299.8
LC	41	36	6.5	86.65	41.5
LC	48	30	6.5	175.54	17.1
LC	50	73	17.4	82.31	88.7
LC	52	59	6.5	135.19	43.6
LC	55	3	6.5	86.8	3.5
LC	56	59	6.5	52.49	112.4
LC	57	33	8.8	159.62	20.7
LC	58	749	351.1	81.66	917.2
LC	59	80	6.5	124.79	64.1
LC	60	n.d	6.5	71.38	n.d
LC	63	94	9	183.76	51.2
LC	43	683	6.5	55.18	1237.1
LC	35	327	7	93.19	350.5

LC	42	1410	14.6	173.91	810.9
LC	36	547	20.3	139.19	392.9

Table S2. Mean vale of urinary pRKIP as well as proteinuria and urinary creatinine recorded for each patient enrolled in phase 1.
