# Supplementary material for: Circulating RKIP and pRKIP in Early-Stage Lung Cancer: Results from a Pilot Study
Source: J Clin Med. 2024 Sep 29;13(19):5830. doi: 10.3390/jcm13195830 (PMC11476948; doi:10.3390/jcm13195830)
Supplement: Supplementary file 1 [file jcm-13-05830-s001.zip › Table S3.pdf]

SAMPLE ID	GROUP	Mean RKIP (µg/mL)	Mean pRKIP (ng/mL)	RpR score
**26**	**HS**	110.1	192.1	573.1
**35**	**HS**	90.4	150.1	602.1
**36**	**HS**	90.5	187.9	481.8
**37**	**HS**	99.6	145.2	685.8
**38**	**HS**	91.9	185.7	494.9
**39**	**HS**	100.5	208.9	481.1
**40**	**HS**	92.4	154.1	599.6
**41**	**HS**	108.3	186.6	580.3
**42**	**HS**	114.4	209.2	546.9
**43**	**HS**	91	107.8	844.5
**44**	**HS**	96.5	215.6	447.6
**45**	**HS**	116.1	220.0	527.8
**46**	**HS**	106.2	109.5	969.6
**47**	**HS**	100.3	168.2	596.5
**65**	**HR-HS**	117.9	71.3	1653.4
**66**	**HR-HS**	105.4	107.8	978.2
**67**	**HR-HS**	116.4	90.6	1284.6
**68**	**HR-HS**	110.1	174.0	632.7
**70**	**HR-HS**	92.6	76.0	1218.3
**71**	**HR-HS**	93.1	184.1	505.7
**72**	**HR-HS**	109.5	139.2	786.6
**73**	**HR-HS**	117.3	85.3	1375.3
**74**	**HR-HS**	93.1	150.9	616.8
**75**	**HR-HS**	126.9	151.7	836.3
**76**	**HR-HS**	141.6	141.0	1004.5
**77**	**HR-HS**	117.3	121.6	964.5
**78**	**HR-HS**	103.4	68.3	1513.9
**79**	**HR-HS**	108.9	160.6	678.2
**80**	**HR-HS**	114.7	141.4	811.2
**82**	**HR-HS**	104	202.6	513.4
**83**	**HR-HS**	102.8	86.8	1183.7
**84**	**HR-HS**	116.2	91.3	1272.1
**85**	**HR-HS**	113.6	136.1	834.7
**86**	**HR-HS**	99.6	102.2	974.5
**87**	**HR-HS**	119.7	148.1	808.1
**88**	**LC**	118.9	92.1	1291.4
**89**	**LC**	112.2	220.5	508.9
**90**	**LC**	118	154.9	761.9
**91**	**LC**	147.4	120.1	1227.8
**92**	**LC**	117.1	155.3	754.2
**93**	**LC**	112.4	126.7	887.4
**94**	**LC**	136.5	130.1	1049.6

**95**	**LC**	116	172.3	673.2
**96**	**LC**	107.9	105.9	1018.5
**97**	**LC**	112.7	201.7	558.7
**98**	**LC**	136.6	202.0	676.2
**99**	**LC**	129.9	154.1	842.9
**100**	**LC**	145.8	227.2	641.7
**101**	**LC**	146.9	144.0	1020.4
**104**	**LC**	115.4	112.4	1026.6
**105**	**LC**	109	135.2	806.3
**69**	**LC**	105.4	121.1	870.4
**81**	**LC**	116.2	110.7	1049.7

Table S3. Mean values of serum RKIP, pRKIP and RpR score recorded by indirect ELISA for each patient enrolled in phase 1.
