# Supplementary material for: Circulating RKIP and pRKIP in Early-Stage Lung Cancer: Results from a Pilot Study
Source: J Clin Med. 2024 Sep 29;13(19):5830. doi: 10.3390/jcm13195830 (PMC11476948; doi:10.3390/jcm13195830)
Supplement: Supplementary file 1 [file jcm-13-05830-s001.zip › Table S4.pdf]

SAMPLE ID	GROUP	mean value RKIP (µg/mL)	median pRKIP (µg/mL)	RpR score
**35**	**HS**	1.4	0.800	1.8
**37**	**HS**	1.9	1.110	1.7
**38**	**HS**	2.4	1.016	2.4
**39**	**HS**	1.3	0.835	1.6
**41**	**HS**	2.3	1.055	2.2
**42**	**HS**	4.7	1.411	3.4
**43**	**HS**	1.8	1.110	1.6
**44**	**HS**	3.3	2.177	1.5
**45**	**HS**	2.2	1.102	2.0
**46**	**HS**	2.9	0.195	14.9
**47**	**HS**	2.1	1.600	1.3
**67**	**HR-HS**	26.9	1.456	18.5
**68**	**HR-HS**	28.4	0.936	30.3
**70**	**HR-HS**	1.8	0.718	2.5
**71**	**HR-HS**	1.9	0.992	1.9
**72**	**HR-HS**	2.8	0.418	6.7
**73**	**HR-HS**	28.2	0.883	32.0
**74**	**HR-HS**	2.6	0.952	2.8
**75**	**HR-HS**	10.8	0.710	15.2
**76**	**HR-HS**	70.8	0.721	98.3
**77**	**HR-HS**	30.0	1.878	16.0
**78**	**HR-HS**	27.6	0.670	41.2
**79**	**HR-HS**	10.4	0.896	11.6
**80**	**HR-HS**	4.9	0.649	7.6
**82**	**HR-HS**	3.7	1.498	2.5
**83**	**HR-HS**	20.8	0.192	108.6
**84**	**HR-HS**	8.7	1.204	7.2
**85**	**HR-HS**	14.5	1.312	11.1
**86**	**HR-HS**	3.4	0.892	3.8
**87**	**HR-HS**	19.4	0.871	22.3
**65**	**HR-HS**	5.8	0.800	7.2
**66**	**HR-HS**	5.9	1.800	3.3
**90**	**LC**	32.4	0.767	42.2
**91**	**LC**	27.6	0.168	164.3
**92**	**LC**	28.0	0.599	46.8
**93**	**LC**	36.6	0.982	37.3
**94**	**LC**	27.6	0.851	32.5
**95**	**LC**	28.0	0.801	35.0
**96**	**LC**	9.6	0.099	96.8
**97**	**LC**	5.9	0.193	30.9
**98**	**LC**	33.4	1.291	25.8

**99**	**LC**	27.1	1.112	24.4
**100**	**LC**	29.2	1.021	28.6
**101**	**LC**	31.1	0.819	38.0
**104**	**LC**	29.4	0.064	462.3
**105**	**LC**	37.1	0.100	370.7
**69**	**LC**	1.0	0.565	1.7
**81**	**LC**	28.2	0.422	67.0
**88**	**LC**	8.8	0.736	12.0
**89**	**LC**	3.9	0.400	9.7

Table S4. Mean values of serum RKIP, pRKIP and RpR score recorded by sandwich ELISA for each patient enrolled in phase 1.
